# In *C. elegans*, High Levels of dsRNA Allow RNAi in the Absence of RDE-4

**DOI:** 10.1371/journal.pone.0004052

**Published:** 2008-12-29

**Authors:** Jeffrey W. Habig, P. Joseph Aruscavage, Brenda L. Bass

**Affiliations:** 1 Department of Biochemistry and Howard Hughes Medical Institute, University of Utah, Salt Lake City, Utah, United States of America; 2 Crowley Davis Research, Eagle, Idaho, United States of America; Victor Chang Cardiac Research Institute, Australia

## Abstract

*C. elegans* Dicer requires an accessory double-stranded RNA binding protein, RDE-4, to enact the first step of RNA interference, the cleavage of dsRNA to produce siRNA. While RDE-4 is typically essential for RNAi, we report that in the presence of high concentrations of trigger dsRNA, *rde-4* deficient animals are capable of silencing a transgene. By multiple criteria the silencing occurs by the canonical RNAi pathway. For example, silencing is RDE-1 dependent and exhibits a decrease in the targeted mRNA in response to an increase in siRNA. We also find that high concentrations of dsRNA trigger lead to increased accumulation of primary siRNAs, consistent with the existence of a rate-limiting step during the conversion of primary to secondary siRNAs. Our studies also revealed that transgene silencing occurs at low levels in the soma, even in the presence of ADARs, and that at least some siRNAs accumulate in a temperature-dependent manner. We conclude that an RNAi response varies with different conditions, and this may allow an organism to tailor a response to specific environmental signals.

## Introduction

The RNA interference (RNAi) and micro-RNA (miRNA) pathways employ small RNAs to modulate gene expression [1]. In both pathways the small RNAs are ∼21–25 nucleotides in length and are processed from dsRNA precursors by the RNase III enzyme Dicer. While some organisms encode multiple Dicer enzymes that function specifically in one pathway or the other, *H. sapiens* and *C. elegans* have a single Dicer enzyme that generates both siRNA and miRNA.

**Figure 1:**
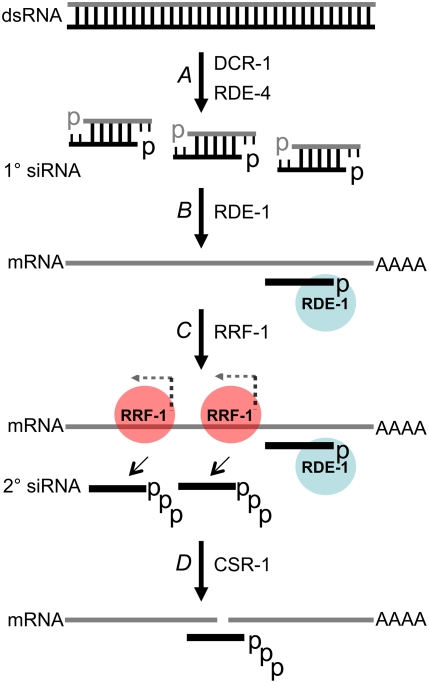
siRNA-mediated silencing in *C. elegans*. (A) dsRNA consisting of a sense (gray) and antisense (black) strand, is processed into primary (1°) siRNAs by the RNase III enzyme DCR-1, in concert with the dsRBP, RDE-4. As is typical of products of RNase III enzymes such as DCR-1, 1° siRNAs are double-stranded, with a monophosphate (p) at the 5′ terminus and a hydroxyl at the 3′ terminus, which overhangs the duplex by two nucleotides; each strand is ∼23 nucleotides long. *In vitro* studies show that human Dicer prefers to cleave from one end of the dsRNA substrate [31], and although not definitively proven, experiments with cell-free extracts indicate *C. elegans* DCR-1 acts similarly [32]. Thus, the three 1° siRNAs shown from left to right represent successive cleavage from the end of the duplex. (B) The Argonaute protein RDE-1 escorts one strand of the primary siRNA to its target mRNA which contains a complementary sequence. (C) The RNA-dependent RNA polymerase, RRF-1, is recruited to the target message where it synthesizes secondary siRNAs that are antisense to the mRNA. The siRNAs are generated by *de novo* synthesis, are 21–22 nucleotides in length, and contain a 5′ triphosphate (ppp). (D) The Argonaute protein CSR-1 promotes cleavage of mRNAs that are base-paired to the secondary siRNA.

**Figure 2:**
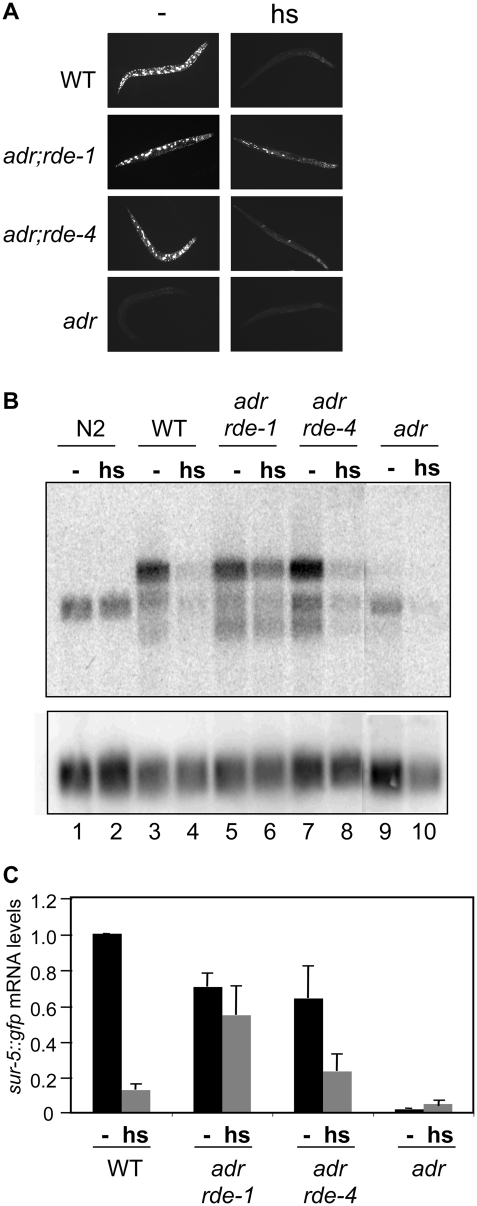
Expression of the GFP transgene in WT and RNAi defective strains, in the presence and absence of heat shock. (A) GFP fluorescence of various strains containing the transgene (uuIs1) was monitored in adult worms grown at 20°C, without (−) or with (hs) heat shock treatment. (B) The northern blot shows hybridization to poly(A+) RNA isolated from adult worms (strains as indicated) that were untreated (−) or heat shocked (hs). All strains carried the transgene except N2, the wildtype parental strain, which served as a control for migration of endogenous *sur-5* mRNA. Membranes were probed for *sur-5* (top panel) or the *gpd-3* loading control (lower panel). The upper band (top panel) present in transgenic lines represents full-length *sur-5::gfp* mRNA. The middle band represents the mRNA originating from the endogenous *sur-5* gene. The lower band is likely a spliced variant of the *sur-5::gfp* transgene as it is detected by probes specific to *sur-5* and *gfp* coding sequence, and also present in an independent strain (SM475) containing the *sur-5::gfp* transgene, but lacking *P_hsp16-2_::GFP(IR)* (data not shown). (C) Bands from multiple northern analyses as in (B) were quantified (see Materials and Methods). Bar height shows the average *sur-5::gfp* mRNA level relative to untreated (−) WT animals, for various strains cultivated without (−) or with heat shock (hs). All samples were normalized to the *gpd-3* loading control; error bars indicate the standard error of the mean (SEM; n = 3–6). *sur-5::gfp* mRNA levels were significantly different (P≤0.04) between untreated and heat shocked samples for all strains except *adr;rde-1* (P = 0.21) and *adr* (P = 0.15); t-test, one-tailed, equal variance.

**Figure 3:**
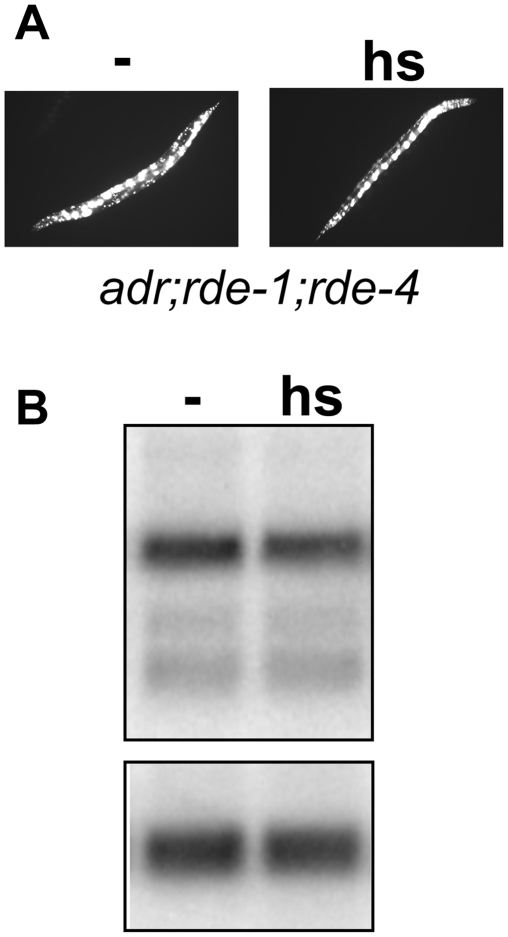
Silencing in *adr;rde-4* depends upon a functional copy of RDE-1. (A) GFP expression in *adr;rde-1;rde-4* adult worms grown at 20°C is shown for untreated (−) and heat shocked (hs) animals. (B) Northern analysis of poly(A+) RNA isolated from *adr;rde-1;rde-4* transgenic worms that were either untreated (−) or heat shocked (hs). Membranes were probed for *sur-5* (upper panel) or the *gpd-3* loading control (lower panel). A statistically significant difference was not observed between untreated and heat shocked samples (n = 3; P = 0.47, t-test, one-tailed, equal variance).

**Figure 4:**
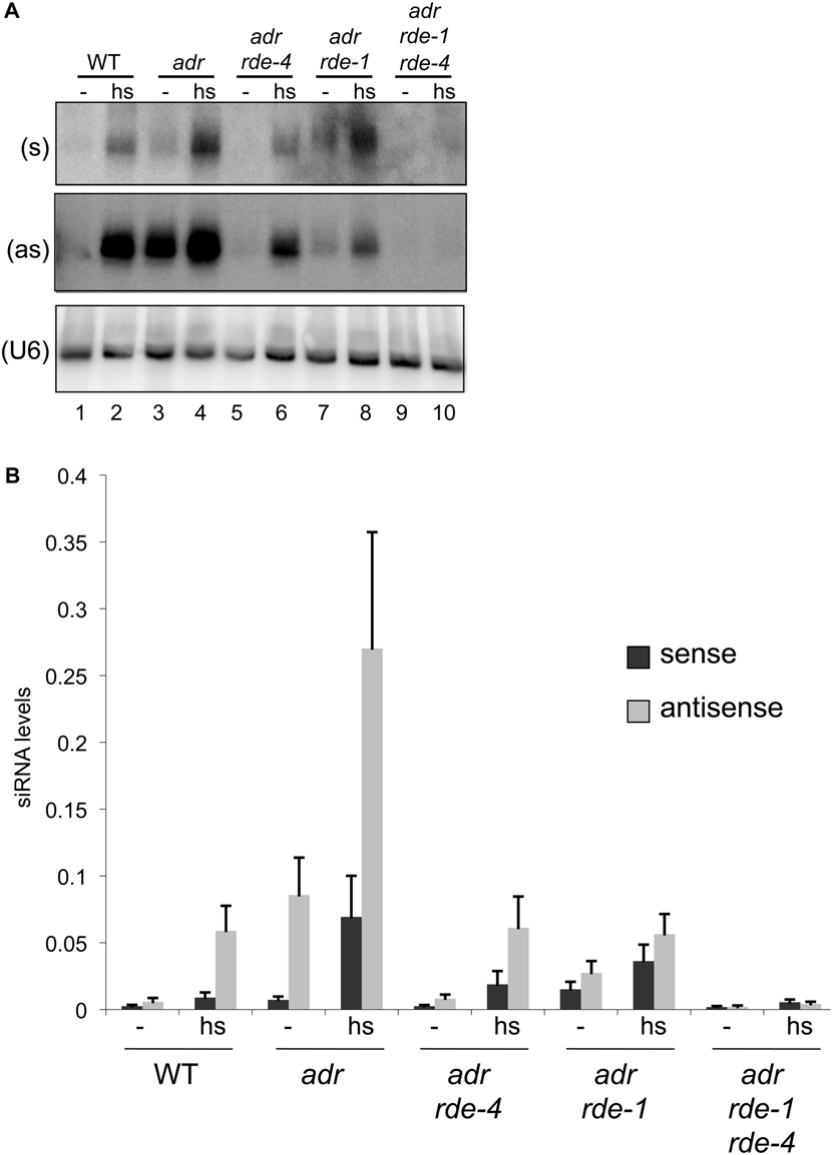
Silencing in *adr;rde-4* animals correlates with siRNA accumulation. (A) Total RNA isolated from adult worms of indicated genotypes (cultivated at 20°C), without (−) or with heat shock (hs), was analyzed by northern analyses. Sense (s, top panel) and antisense (as, middle panel) GFP siRNAs, and the loading control (U6, bottom panel), were detected using ^32^P-end-labeled DNA oligonucleotide probes. To allow visualization of the less abundant sense siRNA, the top blot (s) was slightly overexposed compared to the middle blot (as). (B) Bands from multiple northern analyses as in (A) were quantified after normalizing to a radiolabeled DNA oligonucleotide loaded on the gel to adjust for differences in exposure time. The plot shows sense and antisense siRNA levels calculated as the ratio of GFP siRNA to U6; error bars indicate the SEM (n = 3–6). Various datasets were evaluated with a student's t-test (two-tailed, equal variance), and relevant p-values are shown in Figure S1.

A number of the factors required for the RNAi pathway in *C. elegans* have been identified. The dsRNA-binding protein (dsRBP) RDE-4 acts with Dicer (DCR-1) to facilitate processing of long dsRNA into primary (1°) siRNAs (Fig. 1; [2]–[4]). The Argonaute protein, RDE-1, interacts with RDE-4 [4], [5], but is not necessary for processing long dsRNA by DCR-1 [3]. Rather, RDE-1 acts downstream of 1° siRNA production to facilitate a sequence specific interaction between the 1° siRNA and targeted mRNA. While not understood in detail, RDE-1 is also required to recruit RRF-1, an RNA dependent RNA Polymerase (RdRP; [6]). RRF-1 amplifies the RNAi response by using the mRNA as a template for producing secondary (2°) siRNAs, which ultimately direct the cleavage of the targeted mRNA by the enzyme, CSR-1 [7].

When DNA is introduced into *C. elegans* to form a transgenic strain, it is covalently linked to form long, repetitive arrays. In addition to “sense” mRNA, the arrays often give rise to antisense transcripts that allow formation of dsRNA, which can trigger silencing by the RNAi pathway [8], [9]. While not yet proven, the antisense RNA may arise by read-through transcription of repeats juxtaposed in a converging orientation, or alternatively, by spurious transcription from a cryptic promoter. In *C. elegans*, silencing of transgenic DNA occurs readily for genes expressed in the germline [10], [11] but less so from genes expressed in the soma. The somatic tissue of *C. elegans* is less susceptible to transgene-induced silencing, at least in part, because of the existence of the RNA editing enzymes called Adenosine Deaminases that act on RNA (ADARs; [8]). ADARs convert A∶U base pairs in dsRNA to the less stable I∶U mismatch, thus shifting the dsRNA: ssRNA equilibrium to effectively decrease the amount of dsRNA. Not surprisingly, dsRNA that is pre-treated with ADAR is inefficient in triggering an RNAi response [12].

In theory, *C. elegans* that lack ADARs should have higher concentrations of dsRNA than wildtype animals. We were interested in the effects of higher than normal concentrations of dsRNA on the RNAi pathway. To this end, we compared transgene silencing in wildtype animals with that occurring in mutant strains lacking all ADAR activity, as well as to that occurring in strains designed to express even higher concentrations of dsRNA. We found that, contrary to previous reports, *rde-4* mutant animals are not completely RNAi defective. Instead, in the presence of high concentrations of trigger dsRNA, *rde-4* animals silence a transgene through an *rde-1* dependent mechanism, that by all criteria, corresponds to the canonical RNAi pathway. We also find that high concentrations of dsRNA trigger lead to increased accumulation of primary siRNAs, consistent with the existence of a rate-limiting step during the conversion of primary to secondary siRNAs. Our studies also revealed that transgene silencing occurs at low levels in the soma, even in the presence of ADARs, and that at least some siRNAs accumulate in a temperature-dependent manner.

## Results

*C. elegans* encode two ADAR genes, *adr-1* and *adr-2*, and our studies employed a double mutant containing homozygous mutations in both genes (herein referred to as *adr*). In addition, both wildtype (WT) and *adr* animals used in our studies contained an identical, integrated transgenic array [8]. The array encoded an endogenous protein, SUR-5, fused to the reporter protein, GFP, driven by the *sur-5* promoter (*sur-5::gfp*). In addition, the transgenic array included the *rol-6* phenotypic marker (pRF4) and an inverted repeat consisting of GFP sequence driven by the *hsp-16-2* heat shock promoter (GFP[IR]). As mentioned, because of their repetitive nature, most transgenic arrays introduced into *C. elegans* produce dsRNA. In addition, the GFP[IR] included in this array allowed additional dsRNA to be synthesized by heat shock.

### Overexpression of dsRNA leads to silencing in *adr;rde-4* worms

As shown previously [8], in the absence of heat shock, GFP expression from the transgenic array is dramatically reduced in *adr* mutants compared to WT animals (Fig. 2A, – panels). The silencing observed in *adr* animals results from dsRNA generated from the transgenic array and is dependent on factors required for RNAi. For example, GFP expression is restored when *rde-1(ne219)* or *rde-4(ne299)* mutant alleles are introduced into the *adr* strain (Fig. 2A, – panels; [8]).

Although GFP expression was clearly rescued in *adr;rde-1* and *adr;rde-4* strains, we considered the possibility that some degree of transgene silencing was occurring in these strains, despite the fact that both RDE-1 and RDE-4 are considered essential for RNAi [13], [14]. We reasoned that if the putative silencing involved dsRNA, the phenotype would be enhanced when the concentration of dsRNA was increased. We tested this idea by inducing GFP[IR] expression in *adr;rde-1* and *adr;rde-4* strains with heat shock. Indeed, there was a dramatic reduction in GFP expression in *adr;rde-4* following GFP[IR] induction (Fig. 2A, – vs. hs; Table 1, 20°C vs. hs). For unknown reasons, the pattern of expression of *sur-5::gfp* was asymmetric in *adr;rde-1* animals, with the majority of animals showing GFP expression predominantly in posterior regions (Fig. 2A, 6A). However, this posterior GFP fluorescence was generally lower after heat shock (Table 1).

**Table 1:** Visual scoring of worms for GFP expression (scale 0-5).

Strain	16°C	20°C	25°C	h.s.
ildtype	4.0±.1	4.0±0.1	3.9±0.1	1.0±0.0
*dr*	0.5±0.0	0.5±0.0	0.5±0.0	0.5±0.0
*Adr;rde-4*	2.4±0.3	3.6±0.4	3.9±0.1	1.4±0.3
*adr;rde-1*	2.2±0.2	3.1±0.4	3.9±0.1	2.3±0.2
*dr;rde-4;rde-1*	4.0±0.1	4.0±0.1	4.0±0.1*	4.0±0.0
*de-1*	4.0±0.1	4.1±0.1	4.1±0.2	4.0±0.0
*de-4*	4.1±0.0	4.1±0.1	4.1±0.1	4.0±0.0

* Values represent average±std; 3≥n≤16, except *, where n = 2.

To verify that the decrease in GFP expression correlated with a loss of mRNA, as expected if the RNAi pathway was involved, we used northern blotting to measure the levels of *sur-5::gfp* mRNA in strains grown with and without heat shock (Fig. 2B). Using a probe specific to *sur-5*, we detected a single band corresponding to endogenous *sur-5* mRNA in wildtype worms lacking the transgenic array (N2), whereas two additional bands were detected in strains containing the transgenic array. The slowest migrating species was mRNA encoding the *sur-5::gfp* translational fusion, and we focused on this band in our quantitative analyses. The fastest migrating species was likely an alternatively spliced product of the translational fusion (see Fig. 2A legend) and paralleled expression of the longer *sur-5::gfp* transcript.

In wildtype animals containing the transgene (WT), heat shock induction of GFP[IR] led to an 87% reduction in *sur-5::gfp* mRNA (Fig. 2B, lane 3 vs. 4; Fig. 2C). The reduction was consistent with the mRNA degradation predicted for RNAi-mediated silencing. Consistent with the reduction but not complete loss of GFP fluorescence when *adr;rde-4* animals were heat shocked (Fig. 2A), a 64% reduction in mRNA levels was measured in these animals (Fig. 2B, lane 7 vs. 8; Fig. 2C). Results for *adr;rde-1* animals were also consistent with assays of GFP fluorescence in that *sur-5::gfp* mRNA levels were slightly lower in the *adr;rde-1* strain after heat shock (Fig. 2B), but a statistically significant difference between the untreated and heat shocked samples was not observed (Fig. 2C).

These findings suggested that when presented with high concentrations of trigger dsRNA, in this case provided by the GFP[IR] transgene, RDE-4 deficient strains were capable of silencing transgene expression through a process that involved loss of target mRNA, as expected in the canonical RNAi pathway.

### Silencing in *adr;rde-4* worms depends on RDE-1

All studies to date indicate that RDE-4 is essential for RNAi in *C. elegans*, and thus we sought further evidence that the GFP silencing observed in the *adr;rde-4* strain involved the canonical RNAi pathway. We reasoned that, if the silencing was due to canonical RNAi, loss of a second RNAi factor that acted downstream of RDE-4 would abrogate GFP silencing in the *adr;rde-4* strain. Previous work indicates RDE-1 acts downstream of RDE-4 in the RNAi pathway [3], [6], and thus, we introduced the *rde-1(ne219)* mutation into the *adr;rde-4* strain. *adr;rde-1;rde-4* animals exhibited bright, uniform GFP expression that was unaffected by heat shock (Fig. 3A, Table 1). Consistent with the robust GFP expression, we detected only a minor decrease (∼4%; P = 0.6, Wilcoxon rank sum) in *sur-5::gfp* mRNA levels in this strain following induction of GFP[IR] (Fig. 3B). This result indicated that the silencing observed in *adr;rde-4* animals was dependent on RDE-1, providing further evidence that it occurs via canonical RNAi. The lack of a significant decrease in GFP expression in the *adr;rde-1;rde-4* strain also confirms that the silencing observed in *adr;rde-4* animals (Fig. 2A) was not due to a general heat shock stress response, but due to induction of GFP[IR].

### Secondary siRNAs are produced during silencing in *adr;rde-4* worms

Our studies indicated that in the absence of RDE-4, silencing can occur through the canonical RNAi pathway. To get further support for this idea, we tested whether small RNAs associated with active RNAi were present in our *rde-4* deficient strains. As illustrated in Fig. 1, canonical RNAi involves both primary and secondary siRNAs. Primary (1°) siRNAs arise by cleavage of dsRNA by DCR-1, and thus, have both a sense and antisense strand. Secondary (2°) siRNAs are synthesized by an RNA-dependent RNA polymerase (RdRP; RRF-1, Fig. 1), using the target mRNA as a template, and are distinguished from 1° siRNAs in that they are only one strand, antisense to the mRNA. Furthermore, 2° siRNAs are more abundant than 1° siRNAs, because the RdRP amplifies the siRNA signal. Thus, a normal RNAi response in a wildtype animal gives rise to a mixture of 1° and 2° siRNAs, characterized by low amounts of sense strand (1°) and much greater amounts of antisense strand (predominantly 2°).

We performed northern blot analyses on small RNAs isolated from various strains, either untreated, or heat shocked to induce GFP[IR]. Blots were probed separately for sense or antisense siRNAs using strand-specific radiolabeled DNA oligonucleotides (Fig. 4A). The specific activities of each probe differed slightly, and thus, to allow quantitative comparison of sense and antisense siRNAs, a defined amount of a control radiolabeled DNA oligonucleotide was loaded onto each gel. By normalizing the radioactivity in each band to the radioactivity of the control DNA oligonucleotide we were able to quantify and compare these data (Fig. 4B; Fig. S1).

As expected, high levels of antisense siRNAs with comparatively lower levels of sense siRNAs were detected in WT worms following the induction of GFP[IR] (Fig. 4A, lane 2; Fig. 4B, antisense ∼7-fold higher than sense). Similarly, antisense siRNAs were observed at higher levels compared to sense siRNAs in *adr;rde-4* worms that were silencing GFP in response to induction of GFP[IR] (Fig. 4A, lane 6; Fig. 4B). The accumulation of siRNAs in the *adr;rde-4* strain was dependent upon RDE-1 as siRNAs were nearly undetectable in *adr;rde-1;rde-4* animals (Fig. 4A, B). These results further support the model that in the presence of high concentrations of dsRNA, RDE-4 is not necessary for siRNA production and canonical RNAi.

WT animals treated with heat shock to induce GFP[IR] had a characteristic sense∶antisense siRNA ratio that typifies a normal RNAi response, where antisense siRNAs are more abundant than sense siRNAs (Fig. 4B, WT, hs). Similarly, *adr* worms that were silencing GFP under normal conditions (no heat shock) had a profile of sense and antisense siRNAs similar to that observed in WT worms after heat shock to induce GFP[IR] (Fig. 4B, *adr*, -). In contrast, we observed a much higher accumulation of sense siRNAs after heat shock in both the *adr* and *adr;rde-1* strain. The accumulation of 1° siRNAs in the *adr;rde-1* strain is consistent with the idea that *rde-1* deficient animals cannot pass 1° siRNAs to the next step of RNAi. Further, the levels of sense and antisense siRNAs were roughly equivalent in the *adr;rde-1* animals, as expected if these siRNAs were 1° siRNAs that derived from DCR-1 cleavage of dsRNA. The accumulation of 1° siRNAs in the *adr* strain after heat shock suggests that at the high levels of dsRNA produced under these conditions, a step between 1° and 2° siRNA production is rate-limiting.

### Low levels of silencing occur in soma of wildtype worms

**Figure 5:**
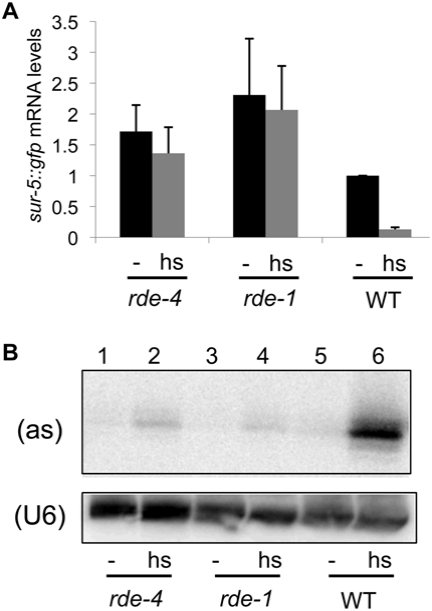
Low levels of silencing occur in the presence of endogenous ADARs. (A) The graph compares *sur5::gfp* mRNA levels derived from the transgene in *rde-4*, *rde-1*, and WT strains in the absence (−) or presence (hs) of heat shock treatment. All strains contain wildtype *adr* alleles. Northern analyses were performed and quantified as in Figure 2. All samples were normalized to the untreated (−) WT reference; error bars indicate the SEM (n = 4–6). Differences between *sur-5::gfp* mRNA levels in untreated and heat shocked *rde-4* and *rde-1* strains were not statistically significant, but both strains showed *sur-5::gfp* mRNA levels (-, hs) that were significantly greater than that of WT (P≤0.07) (B) Northern analysis of small RNAs isolated from adult worms grown at 20°C without (−) or with heat shock (hs). GFP antisense (as, top panel) siRNAs, and the loading control (U6, bottom panel) were detected using end-labeled DNA oligonucleotide probes.

The previous experiments were done in *adr* deficient strains as a means of increasing the amount of unedited dsRNA available to DCR-1 and the RNAi pathway. However, we noticed that we were able to detect low levels of siRNAs from the transgene in our WT strain, where normal levels of endogenous ADAR exist, even without heat shock (Fig. 4A, lane 1). We wondered if the GFP small RNAs detected in the WT strain were entering the RNAi pathway to silence GFP expression. To this end, we introduced the *rde-1(ne219)* and *rde-4(ne299)* alleles into the WT background (hereafter referred to as *rde-1* and *rde-4*, respectively). Indeed, even without heat shock, GFP fluorescence was slightly brighter in *rde-4* and *rde-1* animals compared to wildtype animals (Table 1, 20°C). In addition, even without heat shock, the *sur-5::gfp* message levels were elevated in *rde-4* and *rde-1* animals compared to WT (Fig. 5A, compare - lanes between strains). Further evidence that some transgene silencing was occurring in the presence of ADARs was the appearance of low levels of GFP siRNAs in the WT strain, even in the absence of heat shock (Fig. 5B, WT, - lane). As shown in Fig. 5B, GFP siRNA levels increased in each strain following induction of GFP[IR], consistent with the fact that ADARs are inhibited by high levels of their dsRNA substrate ([15]; see Discussion). These findings suggest that gene expression in wildtype animals is modulated by the RNAi pathway even in the presence of ADARs.

### Rescue of transgene silencing by RNAi factors is temperature sensitive

**Figure 6:**
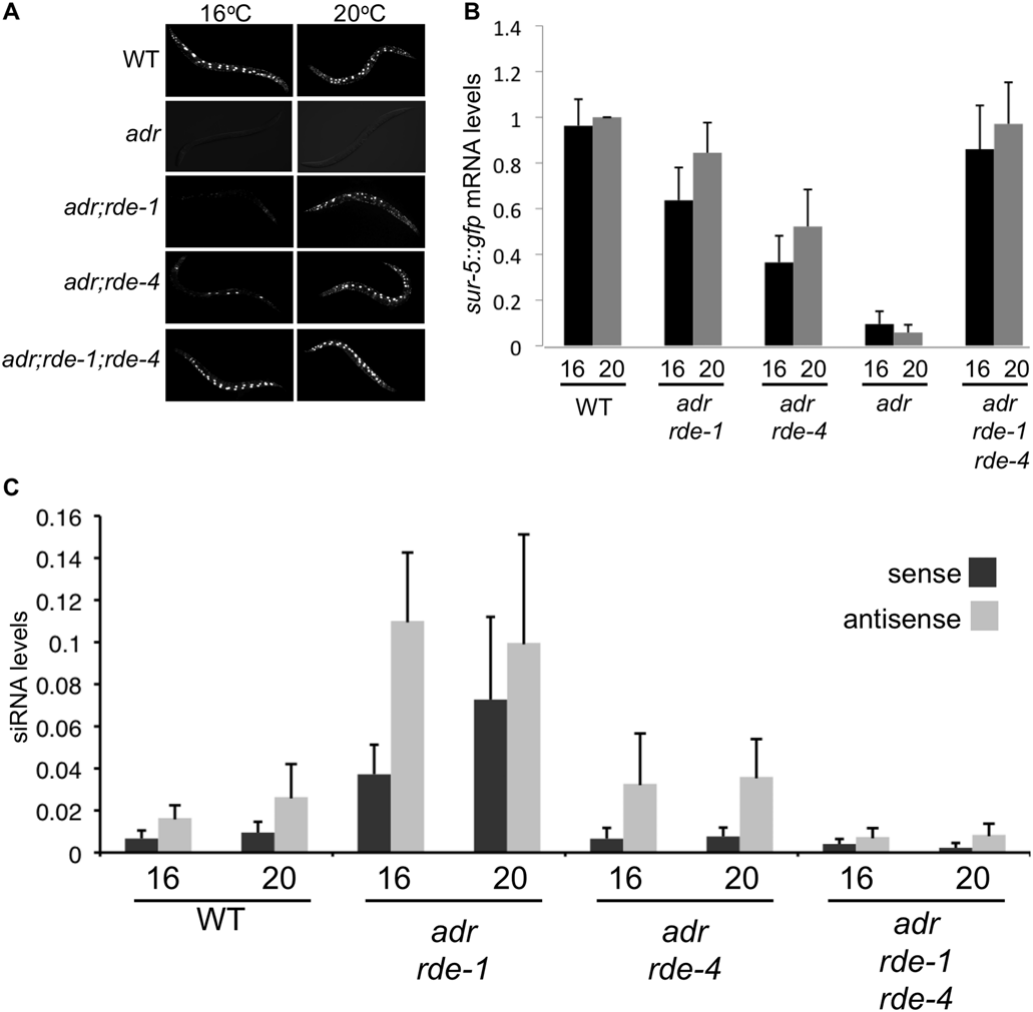
Silencing in *adr;rde-4* and *adr;rde-1* transgenic worms is enhanced at 16°C. (A) GFP expression is compared for adult animals of indicated genotypes grown at 16°C (left) and 20°C (right). (B) Bar height shows *sur-5::gfp* mRNA levels for various strains cultivated at 16°C (black) or 20°C (gray). mRNA levels were determined from northern blots of poly(A+) RNA as in Figure 2, and are represented as the ratio of the *sur-5::gfp* mRNA level to the *gpd-3* loading control, normalized to the WT reference grown at 20°C; error bars indicate the SEM (n = 4). Evaluation by student's t-test showed no significant difference between the levels of *sur-5::gfp* mRNA at 16 and 20°C for any of the strains analyzed. (C) Sense (black) and antisense (gray) GFP siRNAs were quantified from northern blots of RNA isolated from adult animals grown at 16 or 20°C. Data were analyzed as in Figure 4. Bar height shows the ratio of GFP siRNA to U6; error bars indicate the SEM (n = 3–6). Evaluation by student's t-test showed no significant difference between the levels of siRNA at 16 and 20°C for any of the strains analyzed.

As mentioned previously, during our studies we observed variability in the intensity and expression pattern of GFP in the *adr;rde-1* and *adr;rde-4* strains when grown at 20°C; this was not true of any other strains. We considered the possibility that there was an aspect of silencing that was sensitive to small temperature changes. Thus, we compared GFP expression in each strain cultivated at 16 or 20°C. Indeed, while most strains showed a characteristic level of GFP expression that was constant over the temperature range, both *adr;rde-1* and *adr;rde-4* strains showed a decrease in GFP expression as the temperature decreased (Fig. 6A, Table 1). The decrease in expression correlated with only a slight decrease in *sur5::gfp* mRNA (Fig. 6B), and we did not observe a significant difference between the levels of small RNAs at 16°C and 20°C in *adr;rde-1* and *adr;rde-4* animals (Fig. 6C). The discrepancy between the dramatic difference in GFP expression at lower temperatures, and the relatively small changes in mRNA levels, may indicate that our northern analyses are not sensitive enough to detect these small changes. Alternatively, the discrepancy may indicate that some aspect of GFP silencing at 16°C involves inhibition of translation, without loss of mRNA, as would occur if the small RNAs were diverted into the miRNA pathway [16], [17].

### Temperature dependent accumulation of X-cluster siRNAs

**Figure 7:**
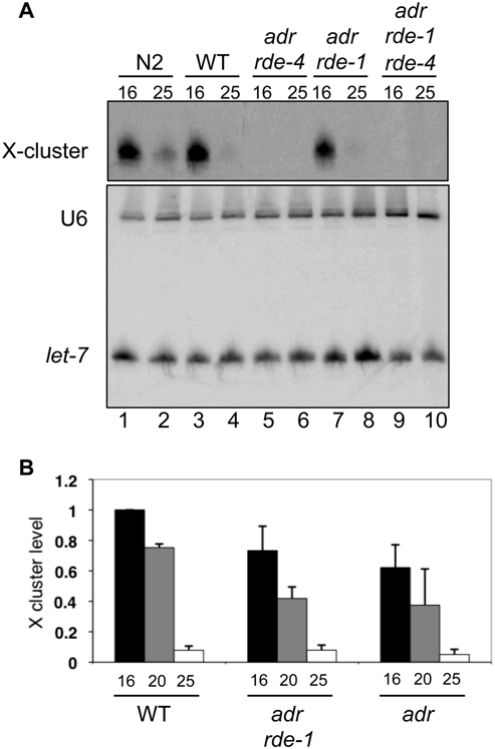
X-cluster endo-siRNAs are more abundant in worms cultivated at lower temperatures. (A) Northern analysis of total RNA isolated from adult worms grown at 16 or 25°C. The X-cluster endo-siRNAs (top panel), loading control (U6, bottom panel) and miRNA (*let-7*, bottom panel) were detected using end-labeled DNA oligonucleotide probes. (B) Northern analyses as in (A) were quantified, and the bar height shows the ratio of X-cluster siRNA to U6 at various temperatures (relative to 16°C); error bars indicate the SEM (n = 3). Data are not shown for *adr;rde-4* and *adr;rde-1;rde-4* strains because X-cluster endo-siRNAs were undetectable in these strains at any of the temperatures monitored (Fig. 7A).

We wondered whether the temperature sensitive accumulation of siRNAs was specific to the dsRNA coming from the array, or if it could be observed for endogenous small RNAs as well. Therefore, we probed our northern blots for the miRNA, *let-7*, and the X-cluster endo-siRNAs, as they are both readily detectable in adult worms. Consistent with previous studies [18], [19], our ability to detect the X-cluster endo-siRNAs was dependent upon RDE-4, and this held true at all temperatures (Fig. 7A, lanes 5–6 and 9–10). However, their accumulation was not dependent upon RDE-1 as high levels of X-cluster endo-siRNAs were observed at low temperatures in *adr;rde-1* (Fig. 7A, lanes 7–8) and *rde-1* animals (data not shown). Furthermore, as reported [20], these endo-siRNAs were strand-specific as we were unable to detect endo-siRNAs at any temperature when probing for the opposite strand (data not shown).

Accumulation of the X-cluster endo-siRNAs was very sensitive to cultivation temperature. While the endo-siRNAs were barely detectable at 25°C, a dramatic increase was observed as the temperature was lowered to 16°C (Fig. 7A,B). The same trend did not apply to the miRNA, *let-7*, (Fig. 7A, bottom panel) indicating the effect was not applicable to all DCR-1 substrates.

## Discussion

### RDE-4 is not necessary for RNAi in the presence of high concentrations of dsRNA

RDE-4 facilitates processing of dsRNA by DCR-1, and *rde-4* mutant animals are defective for RNAi [2], [13], [14]. However, our studies indicate that RNAi occurs in *C. elegans* deficient for RDE-4 if the trigger dsRNA is provided at high concentrations. The transgene silencing we observed in RDE-4 defective worms has attributes of conventional RNAi. Silencing was dependent upon RDE-1, and correlated with decreased mRNA levels and the production of 1° and 2° siRNAs.

If RDE-4 is not essential for silencing via the RNAi pathway, what is its function? RDE-4 is required for DCR-1 cleavage of long dsRNA to siRNA, but is not required for DCR-1 processing of pre-miRNA to miRNA. *In vitro* studies show that RDE-4 preferentially binds long dsRNA [2], [21], and possibly RDE-4 exists to confer this specificity to DCR-1. Since pre-miRNA processing requires only a single DCR-1 cleavage event, one possibility is that RDE-4 increases the affinity of DCR-1 for long dsRNA so that multiple cleavage events can occur before the enzyme dissociates. Alternatively, DCR-1 may be more often bound to the abundant miRNA precursors, and RDE-4 functions to divert DCR-1 from the processing of miRNA precursors to the processing of long dsRNA. This mechanism might be especially useful in responding to long dsRNA associated with viral replication. Consistent with this idea, both *rde-1* and *rde-4* have roles in suppressing VSV replication in *C. elegans*, although to different extents [22].

RDE-4 is necessary for accumulation of certain endo-siRNAs, including the X-cluster (Fig. 7; [18], [19]). The importance of these endo-siRNAs remains unclear as RDE-4-deficient worms develop normally and are fertile. However, our finding that the level of the X-cluster endo-siRNAs is influenced by cultivation temperature raises the possibility that some endo-siRNAs have roles in environmental responses, for example, to down-regulate metabolic processes at lower temperatures. As reported by others, we saw a dependence upon RDE-4 for accumulation of X-cluster endo-siRNAs, and in addition, we found that X-cluster accumulation was not dependent upon RDE-1 (Fig. 7). This suggests that these small RNAs are not entering into the canonical RNAi pathway despite their dependence upon DCR-1/RDE-4. Thus, RDE-4 appears to have multiple cellular roles, one in canonical RNAi and another in the endo-siRNA pathway. It will be interesting to determine if these roles are separable in the cell, such as in cellular localization or through distinct complexes. Alternatively, the separation of the pathways may be dependent upon sorting of siRNAs following processing by the DCR-1/RDE-4 complex.

### Accumulation of primary siRNAs suggests an siRNA amplification step is rate limiting *in vivo*

During an RNAi response, 1° siRNAs are present at low levels relative to the more abundant 2° siRNAs [23], [24]. Using northern blotting to probe for sense and antisense siRNAs independently, on average, we observed ∼12-fold more antisense siRNAs than sense siRNAs in the *adr* strain when grown normally without the heat shock treatment (Fig. 4B). However, when GFP[IR] was induced in this strain by heat shock, the fold increase in sense siRNAs was greater than that for the antisense siRNAs. Following heat shock, antisense siRNAs were on average only 4-fold greater than sense siRNAs. Since sense siRNAs are 1° siRNAs, this result indicates that one of the steps involved in the conversion of 1° siRNAs into 2° siRNAs is rate limiting. Further studies are necessary to determine whether one of the proteins involved in RNAi is limiting or perhaps the mRNA target itself has been consumed in the reaction.

### Keeping silencing at bay

The studies we describe were performed with a transgenic array that encodes GFP as well as a heat shock inducible GFP hairpin. Upon heat shock, dsRNA transcribed from the hairpin enters the RNAi pathway so that GFP expression is silenced. In addition, even without heat shock, low levels of antisense transcripts are produced from this repetitive transgenic array [8]. Presumably, these antisense transcripts hybridize with a fraction of the much more abundant GFP sense mRNA to produce a small amount of GFP dsRNA. Thus, in the absence of heat shock, we observed that WT animals exhibited robust GFP expression, but also showed detectable levels of GFP siRNAs (Fig. 5B). As further evidence that a low level of silencing was occurring in these animals even in the absence of heat shock, *sur-5::gfp* expression from the transgenic array in the WT strain was lower than that of the same array in the *rde-1 or rde-4* mutant background (Fig. 5A, compare - lanes between strains).

Silencing in wildtype animals by the low levels of dsRNA produced in the absence of heat shock was ameliorated, at least in part, by the presence of ADARs. This is evidenced by the observation that, in *adr* mutants, the low level of dsRNA was sufficient to completely silence GFP expression. ADAR enzymes are quite sensitive to substrate inhibition, and at high concentrations of their dsRNA substrate, are catalytically inactive (reviewed in [25]). This is consistent with our observation that in WT animals expressing normal amounts of ADAR, the increased amount of dsRNA produced during heat shock was able to trigger robust silencing.. Of course, it is also possible that the dsRNA binding capability of ADARs, rather than their deaminase activity, is responsible for antagonizing RNAi. In this scenario, ADARs would sequester dsRNA so that it was unavailable to the dsRNA binding proteins involved in RNAi. However, at high concentrations of dsRNA as produced during our heat shock protocol, ADARs would be titrated, allowing the excess dsRNA to enter the RNAi pathway.

Our study with the GFP transgenic array has interesting implications in regard to expression of endogenous transcripts. The data suggest a model whereby there is a constant interplay between mRNA expression and its silencing, dictated by the relative levels of sense and antisense transcripts, as well as by processes that regulate silencing, such as ADARs. Transcriptome profiling indicates many genes give rise to both sense and antisense RNA (see [26], [27]), and we speculate that such genes may be subject to RNAi-mediated regulation.

## Materials and Methods

### *C. elegans* strains

Transgenic strains were generated in the Bristol strain N2. *C. elegans* culture conditions were as previously described [28]. Transgenic lines all include the integrated array uuIs1(sur-5::GFP,pRF4.phsp16::GFP[IR] IV.): WT, BB14 (uuIs1); *adr*, BB (*adr-1(gv6)* I, *adr-2(gv42)* III, uuIs1); *adr;rde-4*, BB109 (*adr-1(gv6)* I, *adr-2(gv42)* III, *rde-4(ne299)* III, uuIs1); *adr;rde-1*, BB111 (*adr-1(gv6)* I, *adr-2(gv42)* III, *rde-1(ne219)* V, uuIs1); *adr;rde-1;rde-4*, BB118 (*adr-1(gv6)* I, *adr-2(gv42)* III, *rde-4(ne299)* III, *rde-1(ne219)* V, uuIs1); *rde-1*, BB107 (*rde-1(ne219)* V, uuIs1); *rde-4* BB112 (*rde-4(ne299)* III, uuIs1).

### Transgenics

The transgenic array previously described [8] was integrated and out-crossed in the N2 background (Scott Knight, unpublished). The array was subsequently crossed into all other backgrounds; genotypes were confirmed by single worm PCR. The *adr;rde-1* and *adr;rde-4* strains were independently generated multiple times to ensure observed phenotypes were not associated with changes in the array during mating.

### Heat shock treatment and culture conditions

Worms were synchronized at the L1 stage by bleaching adults and hatching embryos overnight using standard protocols [29]. Hatched worms were washed in M9 and filtered through Miracloth (Calbiochem) before starting liquid cultures (100 mL S-basal complete with HB101 as food source). Cultures were incubated with shaking at the desired cultivation temperature (16, 20, or 25°C).

Each heat shock was performed by transferring liquid cultures to a shaking water bath preset to 33°C followed by a two-hour incubation. The initial heat shock was performed 8 hours after the addition of food to the synchronized liquid cultures. Subsequent heat shocks (2 through 4) were performed similarly after allowing the cultures 12 hours of recovery at 20°C between heat shocks. The control worms were grown under identical conditions (20°C) but did not undergo the heat shock treatment.

### Visual scoring of GFP

Young adult worms were scored for intensity of GFP fluorescence on a scale of 0 to 5. For each determination, several worms were picked at random and photographed with a compound fluorescence microscope using a constant exposure time. Images were compared to estimate relative GFP expression (Table 1). A baseline of 0.5 was assigned to *adr* worms, which are completely silenced for GFP except for slight expression in neuronal tissues. Numerical values were assigned by successive side-by-side comparisons. In general, brighter neuronal expression and/or very dull non-neuronal expression was scored near 1.0, dull GFP expression in non-neuronal tissues was scored near 2.0, medium fluorescence was scored near 3.0 and bright fluorescence was scored near 4.0. Very bright *sm475* (control) worms were scored as 4.8. For unknown reasons, GFP expression in *adr;rde-1* worms was asymmetric, with posterior expression predominating. *adr;rde-1* worms were scored by monitoring posterior expression.

### Total RNA isolation

Worms were isolated as young adults (3 days of growth for control worms; 4 days of growth for heat shocked worms). Worms were harvested by allowing to pellet by gravity and washing three times with 0.1 M NaCl to remove bacteria. Worms were then vortexed in 4 volumes of Trizol (Invitrogen) and frozen using liquid nitrogen; total RNA was isolated as per the manufacturer's protocol (Invitrogen).

### Northern Blot Analysis

Analysis of *sur-5::gfp* mRNA levels was performed using standard northern blot protocols (1.2% agarose/formaldehyde) on poly(A+) RNA. Poly(A+) RNA was isolated using the Oligotex mRNA Midi Kit (Qiagen), starting with 50–60 µg of total RNA from each sample. In each case the entire poly A+ sample was electrophoresed for northern blot analysis. RNA was transferred to nylon membranes and probed using strand-specific *sur-5* or *gpd-3* probes made using the Strip-EZ RNA T7 kit (Ambion).

Analysis of small RNAs by northern blot was performed by electrophoresing 40 µg of total RNA on a 15–17% polyacrylamide, denaturing gel. RNA was transferred to Hybond-NX membrane (Amersham Biosciences) in 0.5× TBE using a Biorad transfer-cell. Membranes were treated with EDC [1-ethyl-3-(3-dimethylaminopropyl) carbodiimide] for 30 minutes at 60°C to cross-link small RNAs to the membrane [30]. Membranes were hybridized with ^32^P-labeled DNA oligonucleotides (T4 Polynucleotide Kinase) in Ultrahyb Oligo Buffer (Ambion). GFP siRNA were probed using a mix of 10 different DNA oligonucleotides specific to either the sense or antisense GFP sequence. Probing for U6 was performed using a mix of DNA oligonucleotides complementary to U6, whereas probing for *let-7* was performed using a single DNA oligonucleotide complementary to the mature *let-7* sequence. To allow for comparison of sense and antisense levels, all samples were normalized to a radiolabeled DNA oligonucleotide (internal control) loaded alongside the samples on the gel [e.g. ((sense or antisense)/internal control)/(U6/internal control)]. Membranes were exposed and scanned using PhosphorImager cassettes and the STORM (Molecular Dynamics). Visualization and quantification was performed using ImageQuant software (Molecular Dynamics). Blots were stripped with three 20 min washes at 80°C (0.1% SDS/TE) in between each probing, and the absence of radioactivity was verified by scanning.

## Supporting Information

Figure S1 Relevant P-values.
(0.04 MB PDF)
