# Supplementary material for: In C. elegans, High Levels of dsRNA Allow RNAi in the Absence of RDE-4
Source: PLoS One. 2008 Dec 29;3(12):e4052. doi: 10.1371/journal.pone.0004052 (PMC2603325; doi:10.1371/journal.pone.0004052)
Supplement: Figure S1 — Relevant P-values. (0.04 MB PDF) [file pone.0004052.s001.pdf]

	WT-S	WT-AS	WT-S	WT-AS	A-S	A-AS	A-S	A-AS	A4-S	A4-AS	A4-S	A4-AS	A1-S	A1-AS	A1-S	A1-AS	A14-S	A14-AS	A14-S	A14-AS
WT-S		0.31																		
WT-AS																				
WT-S				0.03			0.04								0.05					
WT-AS								0.02												
A-S						0.03	0.09													
A-AS								0.09												
A-S								0.07												
A-AS																				
A4-S										0.13										
A4-AS																				
A4-S												0.15								
A4-AS																				
A1-S														0.28						
A1-AS																				
A1-S																0.33				
A1-AS																				
A14-S																		0.95		
A14-AS																				
A14-S																				0.65
A14-AS																				

Figure S1. Relevant P-values. P-values for differences in GFP siRNAs in comparisons between sense (S) and antisense (AS) siRNAs, or between various strains, untreated (black font) or heat shocked (red font). Strains are indicated as wildtype (WT) or with letters/digits that represent genes containing mutations: *adr*, A; *rde-4*, 4; *rde-1*, 1. P-values were derived from a student's t-test, two tailed, equal variance.
